# Exploring Oesophageal Cancer in Ethiopia: Elevated Incidence in Females and Younger Cases

**DOI:** 10.1002/cnr2.70048

**Published:** 2024-12-12

**Authors:** Girma Mulisa, Tamrat Abebe, Bekele Gutema, Jannatul Mahmuda, Md. Al Amin Khan, Tarik Gheit, Zdenko Herceg, Fazlur Rahman Talukdar

**Affiliations:** ^1^ Department of Microbiology, Immunology & Parasitology Addis Ababa University Addis Ababa Addis Ababa Ethiopia; ^2^ Department of Biomedical Science Adama Hospital Medical College Adama Ethiopia; ^3^ Department of Medical Laboratory Science Arsi University Asella Ethiopia; ^4^ Shahjalal University of Science and Technology Sylhet Bangladesh; ^5^ Toxicology Society of Bangladesh Bangladesh; ^6^ International Agency for Research on Cancer Lyon France; ^7^ Cancer Research UK Cambridge Institute, Li ka Shing Centre, University of Cambridge Cambridge UK; ^8^ Cancer Research UK Cambridge Centre, University of Cambridge, Li ka Shing Centre Cambridge UK

**Keywords:** Ethiopia, gender and age, oesophageal cancer, oesophageal squamous cell carcinoma

## Abstract

**Background:**

Oesophageal cancer is a public health concern in Ethiopia. Identifying the incidence and demographic profile of the two histological subtypes: oesophageal squamous cell carcinoma (ESCC) and oesophageal adenocarcinoma (EAC) are the key steps in recognizing the disease burden and potential aetiopathological associations.

**Aim:**

The aim of this study is to identify the age and gender‐specific incidence patterns of the most common subtype of oesophageal cancer in a high‐incidence area of Ethiopia.

**Methods:**

A retrospective cross‐sectional study from a high‐incidence oesophageal cancer district in Ethiopia identified 630 cases from the pathology registry of nine hospitals. The patient records were carefully reviewed and data on age, gender, tumour location and histological types was systematically compiled. The patient data were retrieved and descriptive statistics were used to generate results.

**Results:**

ESCC subtype, accounted for constituting 500 (79.437%) cases. A gender disparity was observed, with 62.80% of cases occurring in females and 37.20% in males. This distribution of higher female ESCC incidences aligns with previous findings indicating a regional consistency and probable aetiological factor. Furthermore, ESCC incidence peaked at 40–50 years in females, highlighting an age‐related incidence trend. EAC was observed in 67 (51.5%) females and 63 (48.5%) males showing similar prevalence. Spatial analysis revealed that the majority of ESCC cases were located in the lower oesophagus, followed by the middle part, with fewer instances in the upper oesophagus.

**Conclusion:**

This study from Ethiopia identified ESCC as the predominant subtype, with a marked female predominance and age‐related gender disparities. EAC with a lesser proportion identified with consistent spatial distribution patterns in both genders provide valuable insights into the epidemiological landscape of this disease. These findings emphasize the urgency of targeted research to uncover the underlying factors.

## 1 Introduction

Oesophageal cancer is a malignant neoplasm that affects the oesophagus, the muscular tube that carries food from the mouth to the stomach [1]. It is a significant global health problem and one of the leading causes of cancer‐related deaths worldwide [2]. In Africa, oesophageal cancer is a major public health concern, with a high incidence and mortality rate reported in several countries. Incidence rates vary across different regions, with the highest rates found in Eastern and Southern Africa [3]. In Ethiopia, oesophageal cancer is among the top 10 cancer types [4] with a significant increase in the trend of its incidence [5] and in particular the cases are common in Arsi‐ Bale districts of the Oromia regional state of Ethiopia [6, 7, 8].

Oesophageal cancer is typically classified into two main histological subtypes: squamous cell carcinoma (ESCC) and adenocarcinoma (EAC) [9]. ESCC is the most common subtype of oesophageal cancer worldwide, accounting for over 90% of cases in some regions. Conversely, EAC is more common in Western countries, particularly in the United States and Europe [1, 10]. The differences in histological subtypes between African and Western countries are not fully understood but may be related to differences in risk factors, genetics and environmental factors [9].

Several risk factors have been identified for ESCC in Africa, including smoking, alcohol consumption and poor nutrition. In addition, exposure to environmental factors such as high levels of nitrosamines in traditional food preservation methods [1, 9], as well as exposure to aflatoxins from contaminated food, have also been implicated in the development of ESCC in Africa [11]. In some areas of Africa, ESCC has been associated with the consumption of traditional alcoholic beverages, such as kachasu in Malawi, Mozambique and mahewu in Zimbabwe [12].

The high incidence of ESCC in Africa is a major concern, as this histological subtype is often diagnosed at an advanced stage, which limits treatment options and leads to poor outcomes [2, 13]. The lack of awareness and screening programs in many African countries means that many cases of oesophageal cancer go undiagnosed until they are too late for effective treatment. In addition, the high cost of treatment and limited availability of cancer care facilities in many African countries mean that treatment options are often limited. Furthermore, epidemiological data from African countries are crucial as any differences from European or other Western countries can significantly contribute to clarifying the aetiology of oesophageal cancer.

Studies have shown that males are more likely to engage in risk factors associated with the development of ESCC, such as smoking and heavy alcohol consumption, which may partially explain the higher incidence rates in males [3, 10]. In addition, hormonal differences between males and females may play a role in the development of ESCC. For example, oestrogen has been shown to have a protective effect against the development of ESCC, which could explain the lower incidence rates in females in most populations across the world [10, 14]. By understanding the sex‐specific incidence patterns of ESCC and the underlying risk factors, prevention strategies can be developed that specifically target the populations at the highest risk. For example, prevention campaigns should focus on reducing smoking and alcohol consumption in males and increasing awareness of the protective effects of oestrogen in females. Moreover, novel prevention strategies can be developed based on risk‐group investigations and inferences.

In addition, early detection and screening programs could be developed to specifically target populations at the highest risk. However, age and sex‐specific incidence of ESCC varies depending on local‐specific risk factors among countries and within the country of East Africa [15]. It is difficult to establish a temporal relationship and interpret the male‐to‐female ratio in place and time for oesophageal cancer patients by reviewing medical records from tertiary hospitals due to referral biases. Therefore, in this study, we investigated sex‐specific incidence patterns from referral hospitals found in the catchment of the oesophageal cancer high‐incidence district of Ethiopia, in association with other clinical and demographic characteristics that could help to better understand the aetiology of the disease.

## 2 Materials and Methods

### 2.1 Study Design and Setting

**FIGURE 1:**
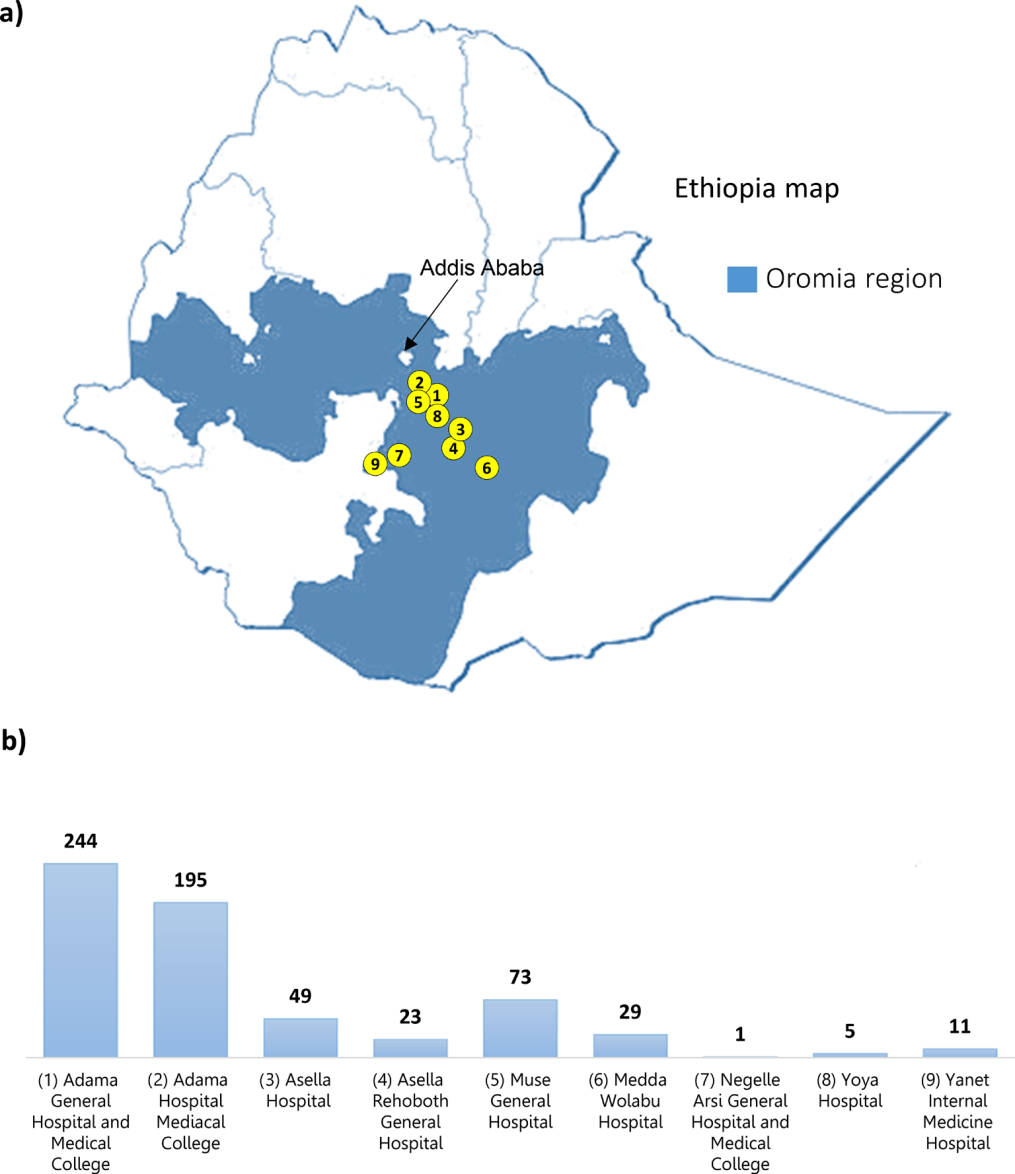
Map of hospital locations included in this study from the Oromia region (coloured in blue) of Ethiopia. (a) Map of selected hospitals from the Oromia regional state in Ethiopia. (b) Names of hospitals and the number of cases enrolled from each hospital.

A healthcare facility‐based cross‐sectional study design was conducted consisting of nine general hospitals namely Adama Hospital Medical College (AHMC), Adama General Hospital and Medical College (AGHMC), Muse Genera Hospital (MGH), Asella Rehoboth General Hospital (ARGH), Medda Wolabu Hospital (MWH), Asella Hospital (AH), Yanet Internal Medicine Hospital (YIMH), Yoya Hospital (YH) and Negelle Arsi General Hospital and Medical College (NAGHMC). The approximate geographic location of these hospitals is shown in Figure 1a. These hospitals were selected as they are located in the catchments of the Arsi‐Bale districts Oromia regional state of Ethiopia where the incidence of oesophageal cancer is high. Due to the availability of endoscopy and pathological examination services, suspected patients are referred to these hospitals from other hospitals in the catchment areas. AH and AHMC provide treatment services with chemotherapy and palliative care for cancer patients.

### 2.2 Data Sources and Study Population

Data from 464 confirmed cases of oesophageal cancer patients from AGHMC, AHMC, and AH were collected retrospectively between August 2019 and August 2022. The data were retrieved from the pathology laboratory registration logbook and patients' records, after obtaining approval from the hospital administration and the oncology department unit. All pathologically confirmed oesophageal cancer cases that were diagnosed during the study period and with complete data with age, sex, histological types and cancer location were included. The remaining patient data from the health care facilities mentioned in the study setting were collected in a prospective manner. The number of cases for which data were collected for each hospital are shown in Figure 1b.

### 2.3 Data Collection Tools and Procedures

A structured data extraction chart was used to collect demographic data (age, sex and residence), and clinical data (oesophageal cancer histological type, anatomical location of oesophageal cancer on the oesophagus and clinical presentation) from both the registration logbook and patients' cards. Oncologic nurses with bachelor's degrees and experience with data collection were recruited for data collection. They were provided with half‐day training covering the purpose of the study and the content of the checklist. Pre‐tests were conducted to familiarise with the data extraction checklist. We acknowledge the possibility of incomplete data collection in some cases. To mitigate this, we implemented a rigorous data verification process and used multiple sources where possible. Cases with significant missing data for the parameters that we were investigating were excluded from specific analyses, and this is noted in the results where applicable.

### 2.4 Data Management and Analysis

Precaution measures were taken to ensure the quality of the data and to avoid duplicate data collected from the pathology laboratory registration logbook and patient card at oncology departments. Descriptive statistics, such as percentages and measures of central tendency and dispersion were computed to describe oesophageal cancer cases by age, sex and cancer location. All demographic characteristics of the patients with oesophageal cancer were presented in the form of figures and tables.

## 3 Results

### 3.1 Patients' Characteristics

Of the 630 oesophageal cancer patients, 381 (60.5%) were female, with a male‐to‐female ratio of 1:1.53. Majority of the patients 398 (63.17%) were below the age of 60 years with 72% of total female patients and 50% male patients within this category. The mean age of the patients was 53.58 with an SD of ± 12.9 with the overall age range of 18–105 years. The mean ages of men and females were 57.33 (SD ± 13.2) and 51.12 (SD ± 12.108), respectively.

### 3.2 Distribution of ESCC and EAC Incidence Patterns

**FIGURE 2:**
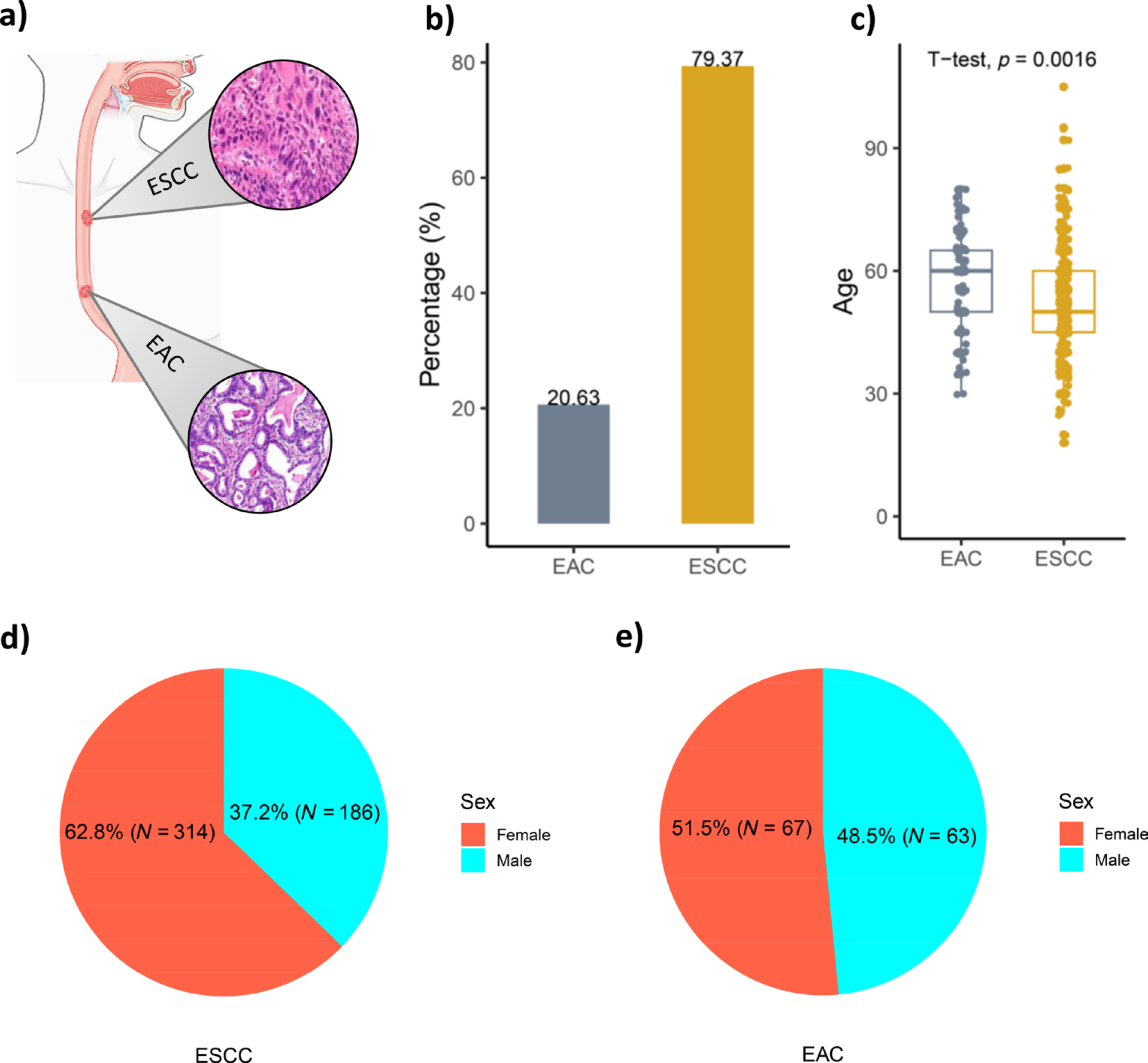
Distribution of the subtypes of oesophageal cancer. (a) The two most common histological types of oesophageal cancer, (b) the proportion of ESCC and EAC in this study, (c) the pattern of ESCC and EAC with the age of the age patients, (d) the proportion of ESCC with respective patients' sex, (e) the proportion of EAC with respective of patient's sex. Abbreviations: EAC, Oesophageal Adenocarcinoma; ESCC, Oesophageal Squamous Cell Carcinoma.

Among the two histological types of oesophageal cancer, ESCC was the dominant type, accounting for 79.37% (*n* = 500) of the total cases (see Figure 2a). In contrast, EAC constituted a smaller, yet noteworthy proportion of 20.63% (130 cases). The distribution of ESCC and EAC, the two histological types of oesophageal cancer in the study population are illustrated in Figure 2b. A higher proportion of individuals with ESCC was observed among younger age groups when compared to those with EAC (Figure 2c).

### 3.3 Sex Distribution Among ESCC Patients

The present study also examined the sex distribution among patients diagnosed with ESCC. The sex distribution among the 500 ESCC patients is shown in Figure 2d, providing insights into the demographic composition of our study participants. This distribution revealed that approximately two‐thirds (314 patients) were females, whereas the remaining third (186 patients) were males. This sex‐specific distribution contributes to an essential component in the larger context of our findings. Unlike the usual ESCC incidence pattern, our study population demonstrated a higher incidence rate among females, accounting for ~62.80% of all cases, whereas males constituted approximately 37.20% of the total cases. The presence of sex‐related differences with increased female incidence not only adds convolution to our findings but also prompts investigations into the potential vulnerabilities associated with females in Ethiopia that may contribute to these divergent phenomena. However, in EAC, the male–female ratio is almost equal with a slightly higher number of females (51.5%) than males (48.5%) (Figure 2e).

### 3.4 Age‐Specific Distribution of ESCC

**FIGURE 3:**
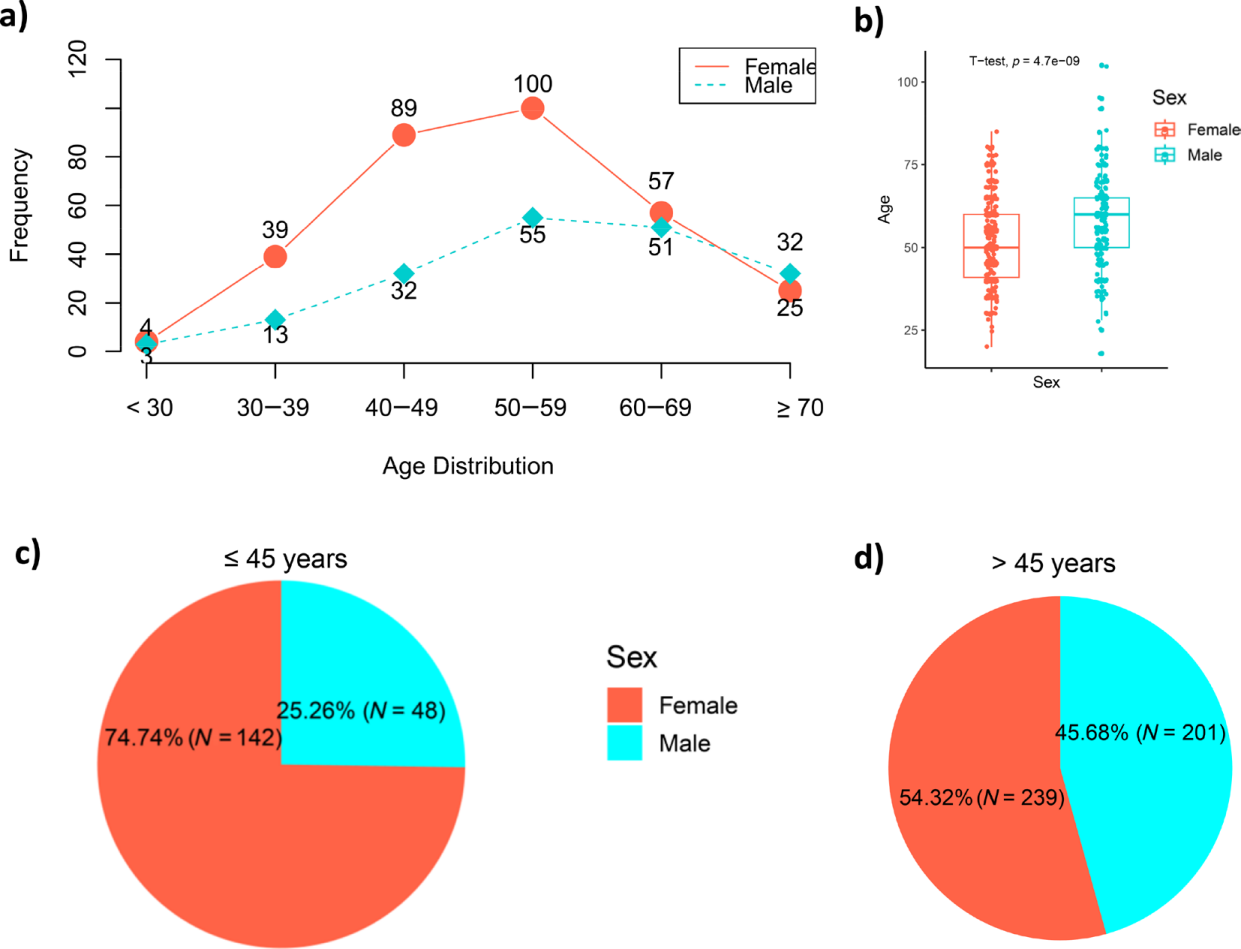
Age distribution between males and females. (a) Comparison of age distribution across various age groups between males and females, (b) mean age distribution between males and females, (c) percentage of individuals with an age of ≤ 45 who have been diagnosed with oesophageal cancer, categorized by gender, (d) percentage of individuals with an age of > 45 who have been diagnosed with oesophageal cancer, categorized by gender.

The analysis of the distribution of ESCC in relation to age revealed that its prevalence varies across different age groups. One notable observation pertains to the substantial disparity in age distribution between males and females within the age range of 30–59 years (Figure 3a). Significantly, there was a notable disparity in the incidence of female ESCC cases within this particular age group, with a prevalence nearly twice as high as that observed among their male counterparts. The average age of individuals with ESCC is notably significantly greater in males than in females (Figure 3b). Moreover, the number of female cases who were ≤ 45 years old was twice as high as the number of male cases in the same age group (as shown in Figure 3c). On the other hand, both sexes had nearly equal proportions of incidence among cases aged > 45 (as shown in Figure 3d).

### 3.5 Anatomical Distribution

**FIGURE 4:**
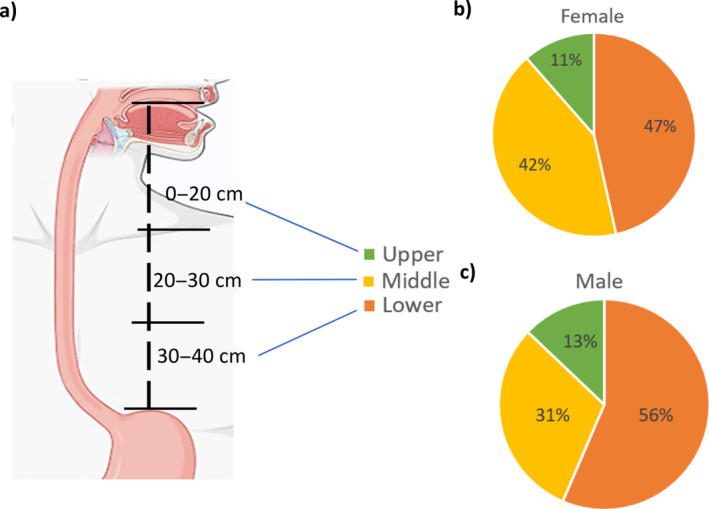
Spatial location of ESCC tumours across the oesophageal. (a) Spatial division of the oesophageal lumen, (b) ESCC cases in females based on the spatial location of the oesophagus and (c) ESCC cases in males based on the spatial location of the oesophagus.

We also investigated spatial distribution patterns of ESCC tumours within the oesophageal anatomy (Figure 4a). These findings offer critical insights into the tumour's preference for specific anatomical segments and contribute significantly to our understanding of preferential locations of ESCC tumours within the oesophagus in our study population. The lower third of the oesophagus has emerged as the epicentre of tumour prevalence, exhibiting a substantial share of ~50.20% of cases. Subsequently, approximately 37.80% of ESCC cases occur in the middle third of the oesophagus. While displaying a slightly reduced prevalence compared to the lower third, this segment's involvement remains a significant component of the ESCC distribution. Interestingly, the upper third of the oesophagus exhibited the lowest incidence, accounting for ~12.0% of all cases. This relatively low prevalence in the upper oesophagus underpins the complex interplay between anatomical factors and tumour initiation. The spatial distribution of ESCC for male and female along the oesophageal anatomy is depicted in Figure 4b,c.

## 4 Discussion

The present cross‐sectional study conducted in Ethiopia provides a significant contribution to the existing literature on oesophageal cancer. Our findings reveal that the incidence rate of ESCC is four times higher than that of EAC. This pronounced discrepancy in the incidence patterns of ESCC and EAC warrants further attention. A recent systematic review and meta‐analysis confirms that the development of ESCC is influenced by a combination of various environmental and lifestyle factors, contributing to the overall risk of the disease [8]. Our findings are consistent with the broader epidemiological context of oesophageal cancer. The elevated incidence of ESCC emphasizes the significance of exploring environmental, genetic, and lifestyle factors specific to the Ethiopian population which could contribute to this prevalence.

In previous research conducted in Ethiopia, it was observed that alcohol consumption and cigarette smoking did not show a significant association with the risk of EC [7, 16]. Conversely, the consumption of hot porridge, intake of dairy products, food cooking practices, x‐ray exposure and khat chewing were identified as having an increased association with EC risk factors [7, 16, 17]. These findings emphasise the necessity for further investigation into the unique associations of these risk factors in association with the diversity of Ethiopia.

Similar findings from Sudan were reported [18, 19] indicating that the same risk factors are present in both settings. However, in Ethiopia, data on the prevalence of ESCC were reported. One study by Deybasso et al. found that almost all (98.3%) of the diagnosed cases were ESCC [8], whereas another study by Wondimagegnehu et al. found that slightly more than half (56.70%) of the cases identified were ESCC [5]. The disparity in numbers can be attributed to the fact that Deybasso et al. collected data from a single district, whereas Wondimagegnehu et al.'s data covers multiple regions of Ethiopia. Geographic variation, which includes differences in lifestyle and environmental exposure, contributes to tumour histological type differences. Similarly, in Pakistan, geographical variation in tumour histological type and location was found [20]. Overall, the low incidence of EAC in the current study could be attributed to predisposing factors for this histological type gastro‐intestinal reflux disease, such as smoking and obesity, which were not previously observed in the catchment population [7, 8] Though our results show a lower proportion of EAC, it is important in feature research for potential aetiological studies because the incidence of EAC is increasing globally [21]. This emphasises the importance of closely monitoring lifestyle changes and other potential aetiologies.

**FIGURE 5:**
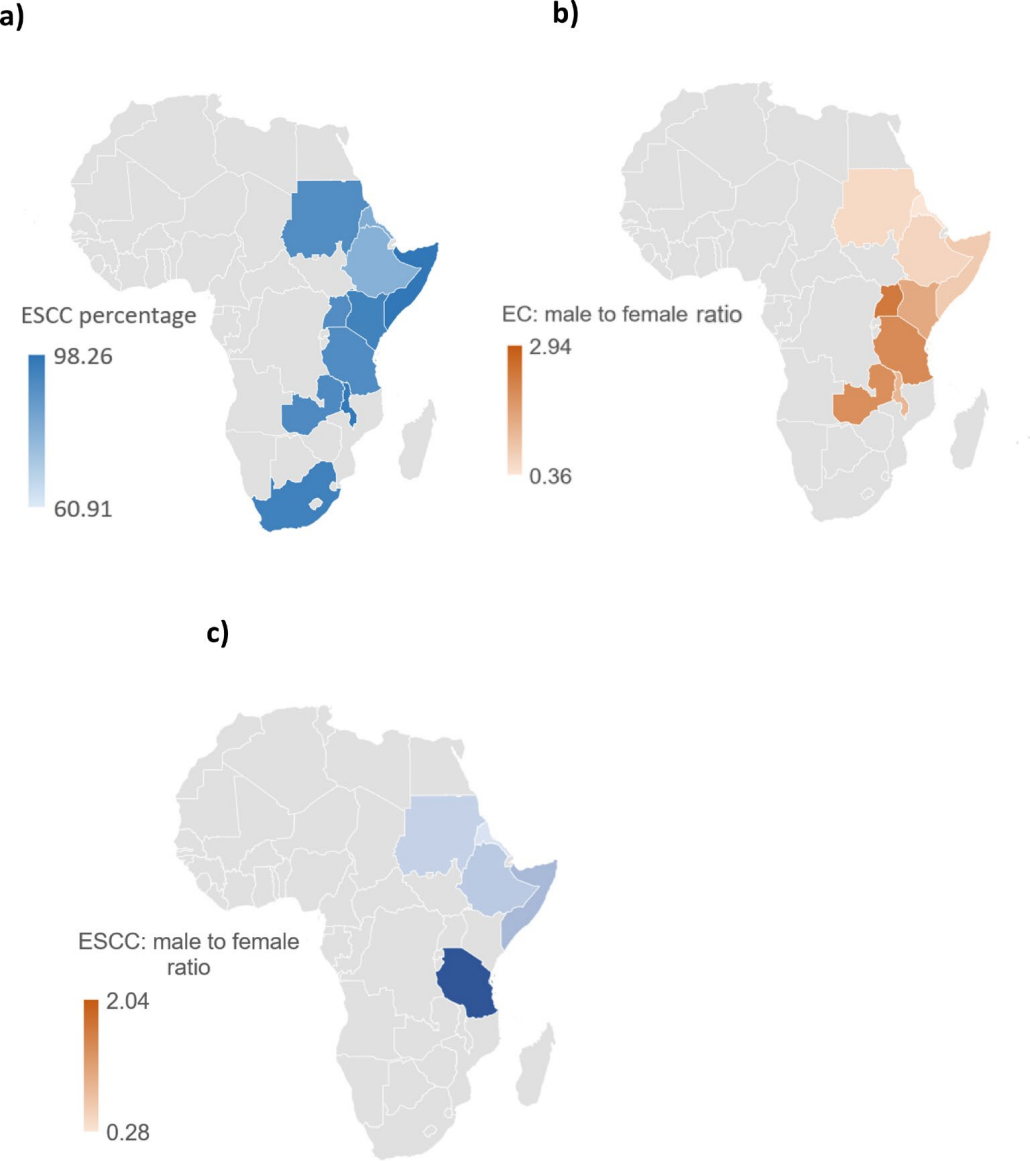
EC distribution across Eastern and Southern Africa: (A) ESCC percentage across Eastern and Southern Africa, (b) male‐to‐female ratio of EC across Eastern and Southern Africa and (c) male‐to‐female ratio of ESCC across Eastern and Southern Africa.

The gender disparity in the incidence of ESCC adds complexity to our understanding of this disease. The higher prevalence of ESCC among women raises important hypotheses regarding the interplay of hormonal, genetic, and behavioural factors. This observation has prompted further investigation into the unique risk profiles of both women and men. Notably, there is a significant gender imbalance in ESCC incidence, with a higher representation of female patients in our cohort. Globally, the incidence of EC is markedly higher in males (70%) than in females [22, 23]. Notably, the incidence of EAC is higher in Ethiopia compared to other countries in the region (Figure 5a), aligning with reports of a global rise in EAC, including in economically disadvantaged countries [21]. In Africa, particularly in Eastern and Southern regions where EC rates are elevated, a significant male predominance in ESCC cases has been reported [3]. For instance, in South Africa, ESCC is more common among males [24]. Similar patterns have been identified in other nations, such as Kenya [25, 26], Tanzania [27] and Uganda [28, 29] where elevated male incidence rates are evident. Conversely, other studies revealed a higher incidence of ESCC among females in Sudan [18, 19], Eritrea [30] and Somalia [31]. Additionally, other studies conducted in Ethiopia have indicated a slightly higher prevalence of female cases [7, 32]. The results of this study were compared with other previous study results from African Oesophageal Cancer Corridor Countries including Sudan, Kenya, Somalia, Uganda, Tanzania, Malawi and Eritrea [33]. Our results were compared with previous studies from Eastern and Southern African countries. These observations are summarized in Table 1 which includes the incidence rates by gender from studies conducted in Ethiopia and neighbouring countries. This comparison reveals that countries in the northwest (Sudan), northeast (Eritrea) and east (Somalia) of Ethiopia exhibit a higher incidence of female cases than males [18, 19, 30, 31]. In contrast, countries to the southwest, such as Kenya, Uganda, and Tanzania, show a two‐ to three fold higher incidence of male cases [25, 27, 28]. The incidence of EC among males steadily increases from East to South Africa, as illustrated in Figure 5b. This geographical‐gender‐based incidence association is also evident in ESCC which is the predominant cancer in this region (Figure 5c). These variations may be attributed to differences in risk factors influenced by geographical and culturally specific activities across countries.

**TABLE 1:** Male‐to‐female ratio of incident oesophageal cancer cases in Ethiopia in comparison to previous research conducted in the neighbouring regions.

Studies	Country	Male‐to‐female ratio		
		Overall oesophageal cancer	ESCC	EAC
Our study	Ethiopia	1: 1.53^a^	1: 1.7^a^	1: 1.06^a^
Wondimagegnehu et al. 2020 Ref‐ [5]	Ethiopia	1:0.80	NA	NA
Deybasso et al.2021 Ref‐ [8]	Ethiopia	1:1.08^a^	NA	NA
Shewaye et al. 2016 Ref‐ [7]	Ethiopia	1:1.24^a^	NA	NA
Gasmelseed et al. 2015 Ref‐ [18]	Sudan	1: 1.76^a^	1:2	2.1
Hamad et al. 2017 Ref‐ [19]	Sudan	1: 1.47^a^	NA	NA
Mengistu et al. 2024 Ref‐ [30]	Eritrea	1:2.78^a^	1:3.48^a^	1:1.06^a^
Kadle and Dufle. 2017 Ref‐ [31]	Somalia	1:1.19^a^	1:1.26^a^	1.85:1
Degu et al.2022 Ref‐ [26]	Kenya	1:0.68	NA	NA
Abdihamid et al. 2024 Ref‐ [25]	Kenya	1:0.94	NA	NA
Gabel et al. 2016 Ref‐ [27]	Tanzania	1:0.49	1:0.49	1:0.54
Obayo et al. 2023 Ref‐ [29]	Uganda	1:0.42	NA	NA
Alema and Iva. 2014 Ref‐ [28]	Uganda	1:0.34	NA	NA

Abbreviations: NA, Not available; Ref, reference.

^a^ Ratios where female incidence > male incidences.

Regarding EAC, our study found a lower incidence compared to ESCC, which contrasts with trends observed in many developed countries. This disparity may stem from lower rates of obesity and gastroesophageal reflux disease in the African population – known risk factors for EAC. Additionally, the tobacco, alcohol, and socioeconomic patterns in our region differ from those in some developed nations, contributing to this difference. Further research is needed to fully understand these differences and their implications for prevention and treatment strategies. To better understand the risk factors associated with ESCC, further research is necessary. Unfortunately, we were unable to gather comprehensive demographic and clinical data, making it difficult for us to comment on the aetiological factors contributing to this pattern of incidence. Specifically, studies should focus on identifying local risk factors that are specific to certain age groups and differentiate between the genders. This information will be invaluable in developing targeted prevention and treatment strategies, enhancing our overall understanding of this disease.

Among women aged less than 45 years, there is a significantly higher incidence rate of ESCC, accounting for 75% of the cases in this age group. This suggests that women below the age of 45 years are more vulnerable to developing ESCC compared to their younger counterparts, or the exposure risk is more common among younger women than among men. In contrast, women aged greater than 45 years exhibited a relatively lower frequency of ESCC, constituting ~57.18% of the cases.

The incidence of ESCC in young people(< 45 years old) was reported previously from Tanzania [34] with infrequent teeth cleaning, second hand tobacco smoke exposure, and pest infestation of grain and/or nuts were identified specific risk factors. In Kenya, significant proportion of young age (< 30 years) were reported to be affected by ESCC where the associated risk factors were not identified [35]. But another study from a case serious report on cases of young age oesophageal cancer risk factors in Kenya reported alcohol and tobacco were not significant predictors of oesophageal cancer (only 15% had exposure to tobacco or alcohol) while the majority (79%) were reported the presence of a family history of cancer [36]. Our findings and the previous report from Eastern Africa warrant further research to identify the potential risk factors of early age onset of ESCC in this region.

These results generate several valuable insights that can be acquired. The observation that ESCC exhibits a higher likelihood of occurrence among women aged 30–59 underscores the necessity of scrutinising sex‐specific risk factors during this specific age group. Variations in hormonal levels and specific lifestyle factors have been identified as potential contributors to the increased susceptibility of women to develop various forms of cancer. Furthermore, our observed findings demonstrate the intricate nature of the aetiology of ESCC in Ethiopia by elucidating the interplay between two major factors such as early age and higher incidence in women which collectively contribute to its divergent patterns of distribution compared to other high‐incidence populations [3].

An increased distribution of ESCC frequency in males with increasing age was observed. Men aged greater than 45 years exhibit a higher incidence rate of ESCC (42%, 82%), whereas men aged less than 45 years demonstrate a lower frequency of ESCC, constituting only 25% of the total cases. These findings highlight a direct age‐related trend in men, indicating that older men are more susceptible to developing ESCC compared to younger men. Patients in Ethiopia often present with advanced stages (III and IV) of the disease, which is compounded by an increased incidence with age until 60 [5]. The high incidence of ESCC among older men predicts a poor treatment prognosis, as advanced age is a predictor of poor treatment outcomes [37]. It is noteworthy that the incidence rates of ESCC in both sexes begin to converge within the age bracket of 60–69 years. This interesting observation implies that, in the context of ESCC, age may exert a greater influence than sex during this specific stage of life, potentially diminishing the significance of sex‐specific factors that were more prominent in earlier stages of life.

The observed sex‐specific variations in the age‐related distribution of ESCC frequency emphasize the complex interplay between age, sex and the development of the disease. The underlying mechanisms driving these differences warrant further investigation. Potential factors contributing to the increased susceptibility of younger women to ESCC may include hormonal factors and early exposure to risk factors. Conversely, the higher ESCC frequency in older males could be influenced by distinct risk factors prevalent in this age group, such as lifestyle choices, dietary habits or long‐term occupational exposures.

The importance of time as a modifier of exposure‐response in chronic disease including cancer risk factors is measured by the age of patients [38, 39]. Understanding the age and sex dynamics in ESCC incidence is crucial for designing targeted prevention strategies, early detection measures and personalized treatment approaches. Tailoring interventions based on age and sex‐specific risk profiles can improve the effectiveness of prevention and management efforts. Furthermore, these findings underscore the importance of age and sex considerations in clinical decision‐making, patient counselling, and public health initiatives aimed at reducing the burden of ESCC. Future research endeavours should focus on elucidating the underlying mechanisms behind these age and sex disparities, paving the way for improved risk stratification and more precise interventions in ESCC management.

The predominant histological type in Africa is ESCC, which is consistent with findings from our and other developing regions [40, 41]. The incidence of EC is higher in males than females across most of Africa, except in North Africa where the rates are similar [42, 43]. This male predominance is particularly evident in high‐risk regions such as Eastern and Southern Africa [43].

In contrast, European countries, especially in the West, have seen a rise in oesophageal adenocarcinoma, largely attributed to lifestyle factors like obesity and gastroesophageal reflux disease. This shift in histological type is less pronounced in Africa, where lifestyle and environmental factors such as tobacco use and low socioeconomic status remain significant risk factors for ESCC [42].

The differences in EC incidence and histological types between Africa and Europe highlight the need for region‐specific prevention and treatment strategies. In Africa, the high mortality‐to‐incidence ratio underscores the challenges in healthcare access and the need for improved diagnostic and treatment facilities [42, 44]. In Europe, addressing lifestyle risk factors could help mitigate the rising incidence of adenocarcinoma. These comparative insights are crucial for tailoring public health interventions to effectively manage and reduce the burden of oesophageal cancer in diverse populations, especially in Africa.

The anatomical distribution of ESCC tumours along the oesophagus provides valuable clinical insights. The concentration of tumours in the lower third of the oesophagus necessitates focused strategies for early detection, particularly in this region. This observation supports the development of tailored screening protocols and therapeutic interventions. The findings also highlight the need for further research to elucidate the underlying factors contributing to the differential tumour distribution within the oesophagus. Factors such as local anatomy, exposure to carcinogens, genetic predisposition and cellular and molecular characteristics may play a role in determining the site‐specific susceptibility to ESCC. Moreover, obtaining valuable information regarding anatomical distribution is crucial for planning potential palliative care, including oesophagostomy, while considering associated complications and patient benefits [45].

Overall, the results emphasize the importance of considering the anatomical distribution of ESCC tumours within the oesophagus in clinical practice and research. Understanding the patterns of tumour occurrence can guide targeted approaches for prevention, early detection and treatment strategies. Moreover, this knowledge may contribute to the development of personalized medicine approaches tailored to the specific needs and risks with tumour location in ESCC patients as tumour location of oesophagael cancer is the predictors of patient survival [37], guide the treatment approach [46] and also the proxy marker of patients' severity of disease (predictors of metastasis) [47].

The results presented here hold implications for clinical practice and public health strategies. Integrating sex and age considerations into screening, diagnosis, and treatment protocols can enhance patient care. The observed disparities also serve as a call for future research aimed at elucidating the underlying mechanisms responsible for the observed trends. Such insights are crucial for the development of targeted interventions, personalized treatments and improved outcomes in the management of oesophageal squamous cell carcinoma.

### 4.1 Strength and Limitation of the Study

This is the largest dataset collected from several hospitals in a region of Ethiopia where oesophageal cancer is common. While our study provides valuable epidemiological data on oesophageal cancer in Ethiopia, we acknowledge that we were unable to thoroughly investigate the causes of observed differences compared to developed countries. This limitation stems from incomplete demographic and clinical data for many patients in our cohort. Future studies with more comprehensive data collection are needed to elucidate the specific aetiological factors contributing to the unique epidemiological profile of oesophageal cancer in Ethiopia and other resource‐limited settings.

## 5 Conclusion

The findings of our study are novel and exciting, urging a need to further investigate the complex mechanisms underlying the age‐ and sex‐related dynamics in ESCC within the Ethiopian population. Understanding the possible causes behind the unexpected incidence of younger age female patients could lead to new methods of early detection, personalized treatment and better outcomes for ESCC patients in Ethiopia. It is essential to improve our ability to fight this severe illness in regions with high rates of occurrence. Our research could introduce new possibilities for the adoption of more effective approaches to prevent and treat ESCC, based on sex and age.

This study is one of the largest cross‐sectional studies conducted in Ethiopia. We identified that ESCC is the most common subtype of oesophageal cancer, accounting for a significant number of cases. Its widespread occurrence has a significant impact on the health landscape of Ethiopia, making it as one of the major health concerns. The marked sex disparity, with a higher incidence among females, not only highlights the need for sex‐specific investigations but also aligns with previous similar findings in Ethiopia and Sudan, hinting at shared underlying factors. Particularly noteworthy is the age‐related trend observed among females, with ESCC peaking at early age < 50 emphasizing the importance of considering age‐specific risk factors in future research and intervention strategies. Spatial analysis revealing most cases in the lower oesophagus further contributes to our understanding of the disease distribution within the Ethiopian context. These findings collectively emphasize the need for a comprehensive, multi‐faceted approach to unravel the complex interplay of factors contributing to ESCC in Ethiopia, providing a foundation for targeted public health initiatives and further research endeavours in this unique population.

## Author Contributions

**Girma Mulisa:** conceptualization, investigation, writing – original draft, methodology, validation, visualization, writing – review and editing, formal analysis, project administration, software, data curation, supervision, resources, funding acquisition. **Tamrat Abebe:** conceptualization, writing – original draft, methodology, visualization. **Bekele Gutema:** project administration, writing – review and editing, writing – original draft, formal analysis, data curation. **Jannatul Mahmuda:** methodology, visualization, writing – review and editing, formal analysis. **Md. Al Amin Khan:** methodology, visualization, writing – review and editing, software. **Tarik Gheit:** methodology, visualization, writing – review and editing, formal analysis. **Zdenko Herceg:** writing – review and editing, methodology, formal analysis, conceptualization. **Fazlur Rahman Talukdar:** conceptualization, investigation, supervision, project administration, formal analysis, methodology, visualization, writing – original draft, writing – review and editing.

## Disclosure

Where authors are identified as personnel of the International Agency for Research on Cancer/World Health Organization, the authors alone are responsible for the views expressed in this article and they do not necessarily represent the decisions, policies, or views of the International Agency for Research on Cancer/World Health Organization.

## Ethics Statement

Ethical clearance of this study was obtained from the Research Ethics Committee of the Department of Microbiology, Immunology and Parasitology and Institutional Review Board of the College of Health Science, Addis Ababa University. Full consent was obtained from the selected patients and hospitals before starting the data collection via a formal letter requesting support from the Department of Microbiology, Immunology and Parasitology. The local institutional review board of the hospitals forwarded a copy of this letter to their respective oncology units/departments for cooperation in the study.

## Conflicts of Interest

The authors declare no conflicts of interest.

## Data Availability

The datasets generated and analysed during the current study are not publicly available due to participant confidentiality, but the electronic version of data are available from the corresponding author on reasonable request.
